# Cholesterol 25-hydroxylase protects against experimental colitis in mice by modulating epithelial gut barrier function

**DOI:** 10.1038/s41598-020-71198-1

**Published:** 2020-08-28

**Authors:** Na Sheng, Zhongnan Ma, Yi Zhou, Juan Xu, Yan Gao, Xin-Yuan Fu

**Affiliations:** 1 The State Key Laboratory of Pharmaceutical Biotechnology, MOE Key Laboratory of Model Animals for Disease Study, Model Animal Research Center, Nanjing University, Nanjing, 210061 China; 2 State Key Laboratory of Biotherapy, Collaborative Innovation Center for Biotherapy, West China Hospital, Sichuan University, Chengdu, 610041 Sichuan China; 3 Deparment of Gynecology, Women’s Hospital of Nanjing Medical University, Nanjing Maternity and Child Health Care Hospital, Nanjing, 210004 China

**Keywords:** Cell biology, Gastroenterology

## Abstract

Cholesterol 25-hydroxylase (CH25H) encodes the enzyme that converts cholesterol to 25-hydroxycholesterol (25-HC). 25-HC has been demonstrated to be involved in the pathogenesis of inflammatory bowel disease. However, the role of CH25H in experimental colitis remains unknown. Dextran sulfate sodium (DSS)-induced colitis was monitored in wild type and *Ch25h*^−/−^ mice in 8-week-old male for 7 days by assessment of body weight, histology, inflammatory cellular infiltration, and colon length. The function of CH25H was investigated using loss-of-function and gain-of-function such as *Ch25h*-deficient mice, supplementation with exogenous 25-HC and treatment of 25-HC into Caco2 and HCT116 colonic epithelial cells. *Ch25h*^−/−^ mice with DSS-induced colitis exhibited aggravated injury, including higher clinical colitis scores, severe injury of the epithelial barrier, lower tight junction protein levels and higher levels of IL-6. Supplementation with exogenous 25-HC ameliorated disease symptoms and reduced the extent of damage in DSS-induced colitis, which was characterized by lower colon damage, higher tight junction protein expression, significantly decreased local and systemic production of pro-inflammatory cytokines IL-6. In Caco2 and HCT116 cells, 25-HC induced tight junction genes expression in colon cancer epithelial cells. These effects of CH25H were obtained by promoting ATF3 expression. Taken together, our findings reveal a protective role for 25-HC in DSS-induced colitis and the ability of CH25H to maintain epithelial gut barrier function through ATF3 expression. Supplementation with exogenous 25-HC ameliorates disease symptoms, which provides a new therapeutic strategy for ulcerative colitis.

## Introduction

Inflammatory bowel disease (IBD), which encompasses Corhn’s disease (CD) and ulcerative colitis (UC), is a chronic inflammatory disorder with increasing incidence and prevalence in many countries^1^. Many genetic and environmental factors have been determined to increase the susceptibility to IBD^2,3^. The main symptoms of IBD include abdominal pain, diarrhea, rectal bleeding and abnormal weight loss^4^, but the mechanisms behind it are less clearly understood. Recent evidence has indicated that the dysregulation of the intestinal epithelial barrier plays a major role in the inflammatory process in IBD^5,6^. Therefore, the underlying mechanisms of inflammation-induced intestinal epithelial barrier damage in IBD need to be elucidated to identify new treatments.

Cholesterol 25-hydroxylase (CH25H) is an enzyme that catalyses the oxidation of cholesterol to form the soluble product 25-hydroxycholesterol (25-HC). 25-HC is an oxysterol that can play an important role in different biological processes^7,8^. Studies of 25-HC have identified it as an innate immune mediator that has antiviral and pro-inflammatory actions, and recent work has identified its anti-inflammatory effect^9,10^. Recent studies have demonstrated that 25-HC exerts antiviral effects against various viruses by inhibiting membrane fusion^10,11^. In addition, 25-HC is able to suppress myelin gene expression in peripheral nerves via LXR/Wnt/β-catenin-mediated pathways in vitro^12,13^. *Ch25h*-deficient mice are viable and behave normally; they do not display any obvious defects under normal conditions^14^. *Ch25h*^−/−^ mice have normal cholesterol homeostasis but exhibit remarkable changes in their inflammatory response^15^. *Ch25h*-knockout mice affects B cell and increases the serum levels of IgA^16^. A lack of *Ch25h* markedly increases the aggressiveness markedly of experimental autoimmune encephalomyelitis (EAE) in *Ch25h*^−/−^/*ApoE*^−/−^ mice, which is a mouse model of multifaceted inflammatory disease^17,18^.

Although the pathogenesis of IBD is complicated, increasing evidence has suggested that IBDs are initiated and developed through the interaction between excess immune reactions and barrier defects in the intestines^19,20^. In DSS-induced colitis, there is an accumulation of activated immune cells, including macrophages, monocytes, dendritic cells, B cells and T cells, which mediates chronic tissue injury through the robust production of inflammatory cytokines^21^. It is known that IL-6, which is secreted by intestinal epithelial cells, plays a critical role in immune cells. Indeed, the mucosal IL-6 level has been reported to be elevated in patients with IBD^22^. On the other hand, the intestinal barrier has an important role in protecting the intestinal. Intercellular tight junctions are located at the junctional region of the intestinal epithelial cells and are essential for maintaining the barrier integrity. Several transmembrane and intracellular proteins such as members of the zonula occludens family (occludin and claudin) and members of the junctional adhesion molecule family, constitute tight junctions^23^. A growing body of evidence has demonstrated morphological and structural changes in tight junctions in IBD patients.

A recent clinical study showed elevated CH25H expression in IBD patients^24^; CH25H was involved in intestinal fibrosis^25^; however, the role of CH25H in IBD has not yet been investigated. To our knowledge, this is the first study showing the ability of 25-HC to alleviate intestinal inflammation in colitic mice. In the present study, we investigated the effect of CH25H on the development of DSS-induced colitis in mice.

## Results

### ***Ch25h***^−/−^ mice are highly susceptible to DSS-induced colitis

**Figure 1:**
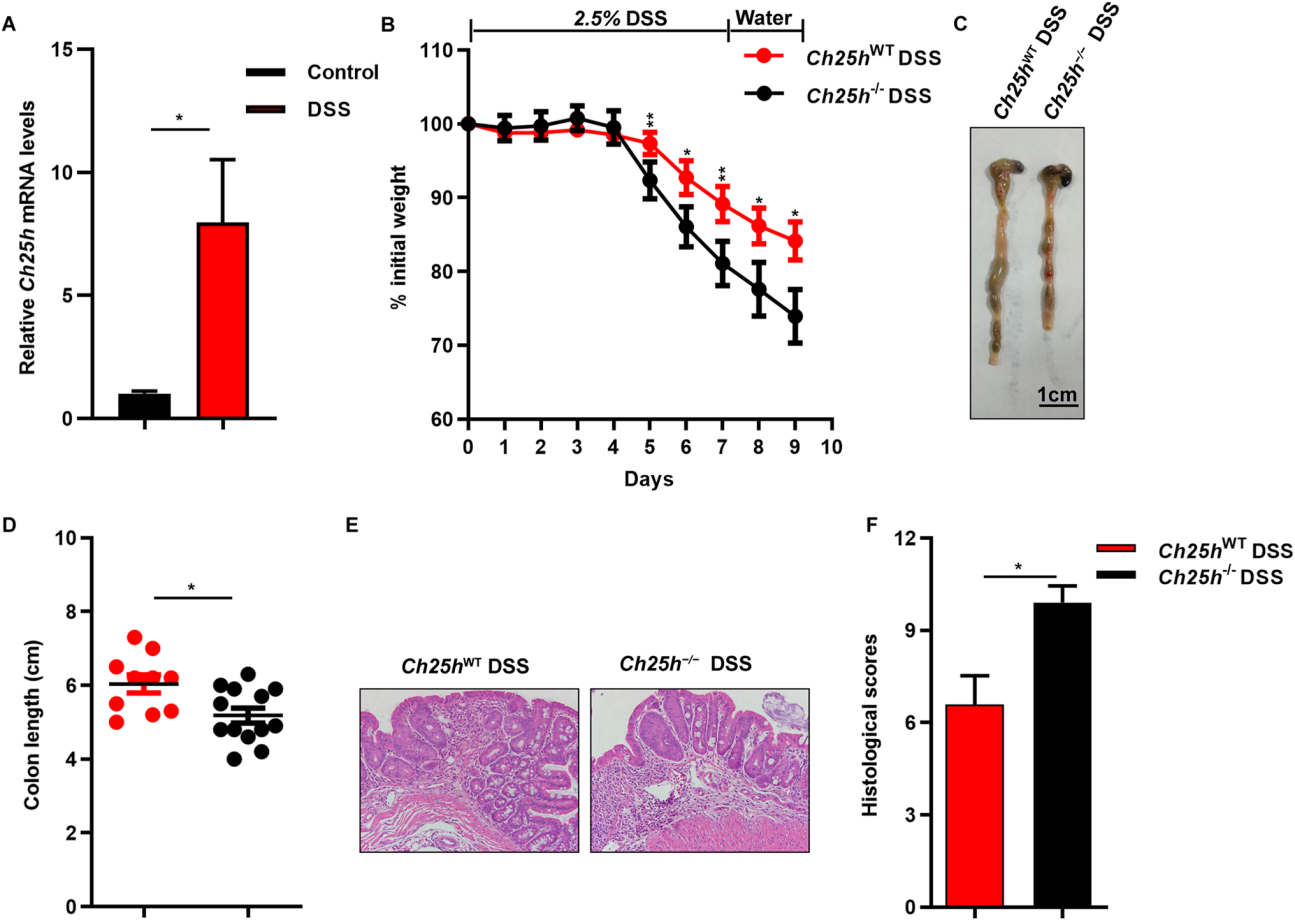
*Ch25h* deficiency increased the efficacy by which DSS induced colitis in mice. (**A**) mRNA expression level of *Ch25h* from colon tissue of WT mice and colitic mice. (**B**) WT and *Ch25h*^−/−^ mice were treated with DSS and body weight is expressed as percent of initial weight. (**C**) Representative images of colons from WT and *Ch25h*^−/−^ mice at day 9. (**D**) Quantification of effect of treatment on colon length. (**E**) Hematoxylin and eosin staining of colon sections from DSS treated WT and *Ch25h*^−/−^ mice at the indicated times. Scale bars, 50 µm. (**F**) Histological scores were determined as described in the section on Material and methods. *p < 0.05, **p < 0.01. Similar results were obtained in three independent experiments with 10–12 mice per group.

Elevated CH25H intestinal tissue levels in UC patients and animal models of acute colitis have been reported to closely correlate with the disease activity^24^. Consistent with previous reports, we observed increased expression levels of CH25H in murine DSS colitis tissue (Fig. 1A). To explore the potential role of CH25H in the pathogenesis of DSS-induced colitis model, we generated the *Ch25h*^−/−^ mice with the CRISPR/Cas9 system (Supplementary Fig. S1A–D). Cyp3a11, Cyp46a1 and Cyp27a1 have been reported to involved in the production of 25-HC^26−28^. Results of the present study showed that deficiency of *Ch25h* did not affect mRNA expression levels of Cyp3a11, Cyp46a1 and Cyp27a1 in the colon (Supplementary Fig. S1D). We investigated the effects of genetic deletion of *Ch25h* on LXR target genes, SREBP processing genes and bile acid synthesis involved in transcription of the *Ch25h* gene^16,18^. mRNA levels of different candidate genes are unchanged in *Ch25h*^−/−^ mice relative to control colon tissue which is consistent with previous report (Supplementary Fig. S1E). *Ch25h*^−/−^ mice developed normally, displayed normal colon length and histological without DSS administration (Supplementary Fig. S2A, B). Then, we administered DSS in drinking water to WT and *Ch25h-*deficient mice, the mice were sacrificed on day 9, *Ch25h*^−/−^ mice exhibited more body weight loss and significantly decreased colon length compared with WT mice (Fig. 1B–D). Histologic analysis showed that *Ch25h*^−/−^ mice developed more severe injury of the epithelial barrier and colonic crypts (Fig. 1E,F). Collectively, these data indicate that *Ch25h*-deficient mice are highly susceptible to DSS-induced colitis.

### ***Ch25h***^−/−^ mice exacerbated DSS-induced inflammation of the colonic mucosa and the activation of the STAT3 signaling pathway

**Figure 2:**
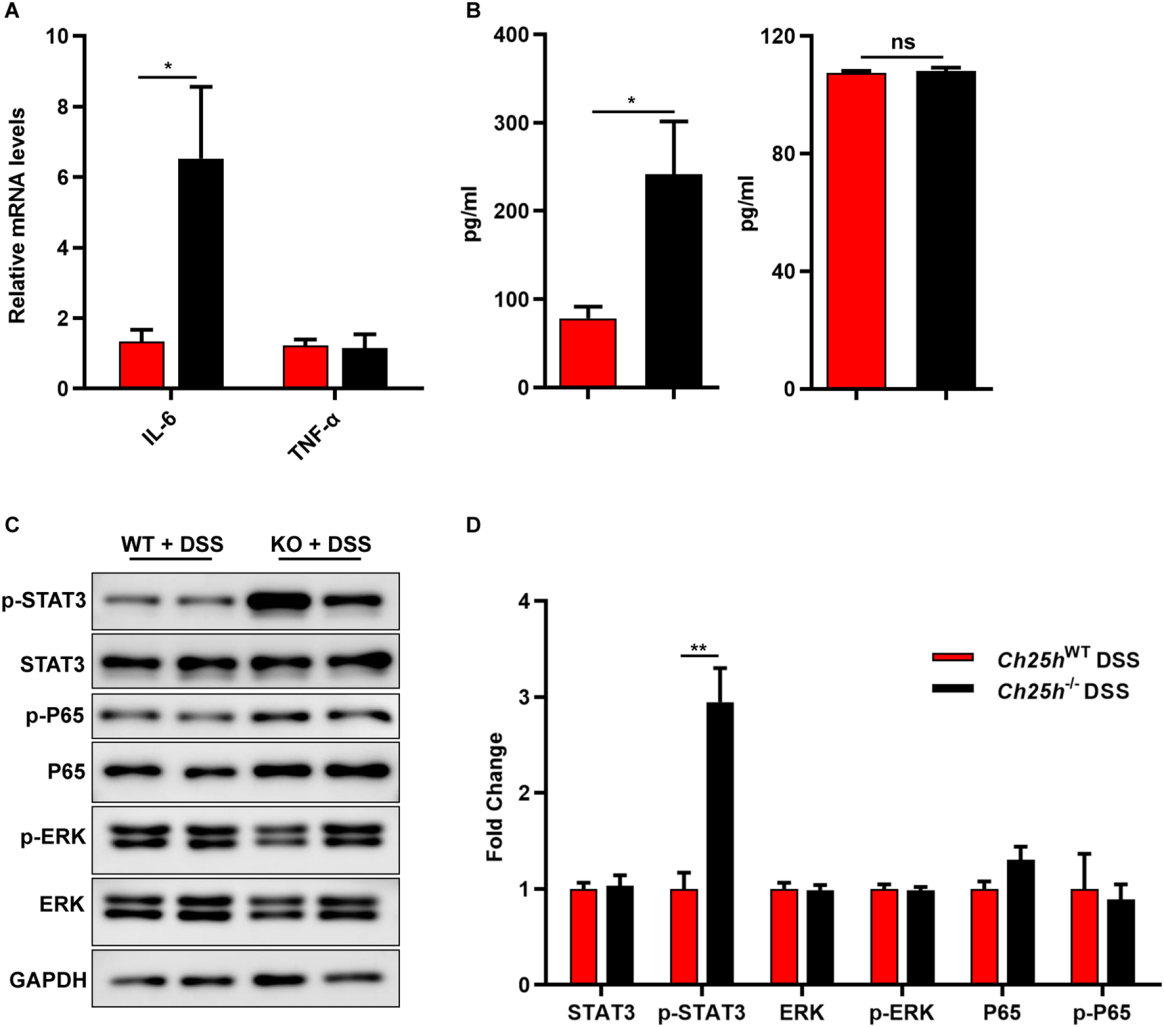
*Ch25h* deficiency exacerbated DSS-induced inflammation of the colonic mucosa and activated the STAT3 signaling pathway. (**A**) The mRNA expression levels of IL-6 and TNF-α in colon tissue were measured by quantitative PCR, and normalized against GAPDH. (**B**) The concentrations of IL-6 and TNF-α in the blood serum of WT and *Ch25h*^−/−^ mice with DSS administration. (**C**) Western blot results of STAT3, ERK, P65 in colon. (**D**) Quantification of the protein level of STAT3, ERK, P65. *p < 0.05, **p < 0.01. The data are expressed as the mean ± S.E.M. Similar results were obtained in three independent experiments with 6–8 mice per group. Full-length blots are included in the supplementary information.

It has been reported that pro-inflammatory cytokines and related factors play an important role in the pathogenesis of colitis, so we analyzed the levels of pro-inflammatory cytokines in DSS-challenged mice. The levels of IL-6, but not of TNF-α, were increased in the colons of *Ch25h*^−/−^ mice relative to control mice after DSS treatment (Fig. 2A). Furthermore, the serum concentrations of IL-6 and TNF-α result were consistent with mRNA levels (Fig. 2B). IL-6 binds to soluble or membrane-bound IL-6 receptors and triggers the activation of STAT3; STAT3 is highly phosphorylated in DSS-induced colitis in mice and human IBD patients^22,29^. In accordance with significantly increased IL-6 levels in the colon, we found an increased phosphorylation of STAT3, but not of ERK or P65, in *Ch25h*^−/−^ mice (Fig. 2C,D). In agreement with these data, we measured the protein levels of phosphorylated STAT3, ERK and P65 and the mRNA expression levels of several pro-inflammatory cytokines and chemokines, including IL-6, IL-1β, TNF-α, Cxcl1, Cxcl2, Cxcl5 and IL-10, in colon tissue from WT and *Ch25h*^−/−^ mice. There were no obvious differences in the absence of DSS administration (Supplementary Fig. S2C, D). Taken together, these results suggest the activation of the IL-6/STAT3 pathway in *Ch25h*^−/−^ mice compared with WT mice upon DSS-induced colitis.

Histological analysis showed an obvious increase in inflammatory infiltrates in DSS-exposed colons of *Ch25h*-deficient mice compared with those of WT mice. To determine the effects of *Ch25h* ablation on immune cellularity, we isolated colon cells from WT and *Ch25h*^−/−^ mice, and analyzed by flow cytometry. As expected, the percentage of CD45^+^ leukocytes was significantly different between the genotypes (Supplementary Fig. S4A). However, our results showed that no obvious difference in the number of T cells between *Ch25h*^−/−^ mice and WT mice in DSS-induced colitis (Supplementary Fig. S4B, C). We next investigated whether *Ch25h* deficiency influences the mucosal infiltration of macrophages by analyzing digested colon tissues by flow cytometric. As expected, DSS treatment led to an increased recruitment of F4/80^+^CD11b^+^ cells (Supplementary Fig. S4D). We also examined the percentages of T cells in the spleen and blood, and flow cytometric analysis revealed that DSS exposure had no effect on T cells in these tissues (Supplementary Fig. S4E, F). Thus, the regulated secretion of pro-inflammation cytokines in the colons of *Ch25h*^−/−^ mice might be due to an increased number of macrophages but not of T cells.

### ***Ch25h***^−/−^ mice exhibit increased damage to intestinal epithelial barrier function

**Figure 3:**
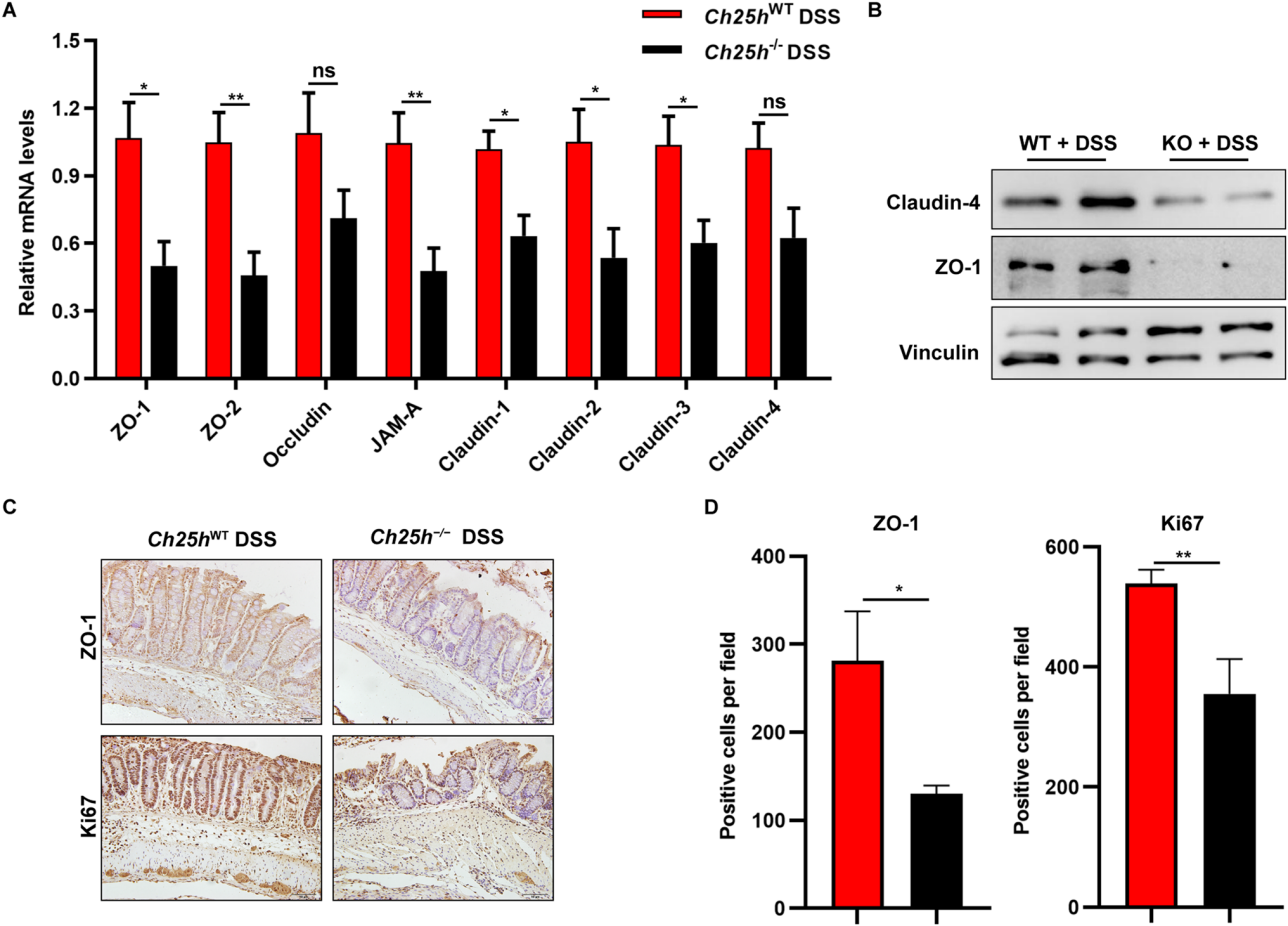
*Ch25h* deficiency increased damage to intestinal epithelial barrier function following DSS treatment. (**A**) mRNA expression levels of ZO-1, ZO-2, Occludin, JAM-A, Claudin-1, Claudin-2, Claudin-3 and Claudin-4 in colon tissue were measured by quantitative PCR, and normalized against GAPDH. (**B**) Western blot results of ZO-1 and Claudin-4 in colon. (**C**) Representative ZO-1 and Ki67 immunostaining of the colon sections from WT and *Ch25h*^−/−^ mice. (**D**) Quantification of ZO-1 and Ki67 positive cells. *p < 0.05, **p < 0.01. The data are expressed as the mean ± S.E.M. Similar results were obtained in three independent experiments with 6–8 mice per group. Full-length blots are included in the supplementary information.

Because a previous report have shown that IL-6-induced STAT3 activation is associated with the reduction of tight junction protein^30^. The RT-PCR analysis showed the mRNA levels ZO-1, ZO-2, JAM-A, Claudin-1, Claudin-2 and Claudin-3 were decreased in colon of *Ch25h*^−/−^ mice. Occludin and Claudin-4 mRNA levels displayed a decreasing trend in DSS-treated *Ch25h*-deficient mice (Fig. 3A). Western blots for ZO-1 and Claudin-2 showed a significant decrease in protein levels (Fig. 3B). In agreement with these data, immunohistochemical analysis showed that ZO-1 was decreased in *Ch25h*^−/−^ mice (Fig. 3C,D). Cell proliferation in the colonic epithelial response to DSS injury is an important factor in regeneration following injury. To examine whether the loss of CH25H might regulates proliferation in the inflamed colon, we analyzed the expression of the proliferation marker Ki67 and revealed a significantly reduced number of Ki67-positive cells in *Ch25h*-deficient epithelial cells compared with WT (Fig. 3C,D). Tight junction expression at the mRNA level was also not significantly different in the absence of DSS administration (Supplementary Fig. S2E). Taken together, results suggest that *Ch25h* deficiency has no effect on the histology or inflammation under normal conditions. These results demonstrate that CH25H is required for intestinal epithelial regeneration and tissue reconstruction in DSS-induced colitis.

### Supplementation with exogenous 25-HC reduced the extent of colon damage

**Figure 4:**
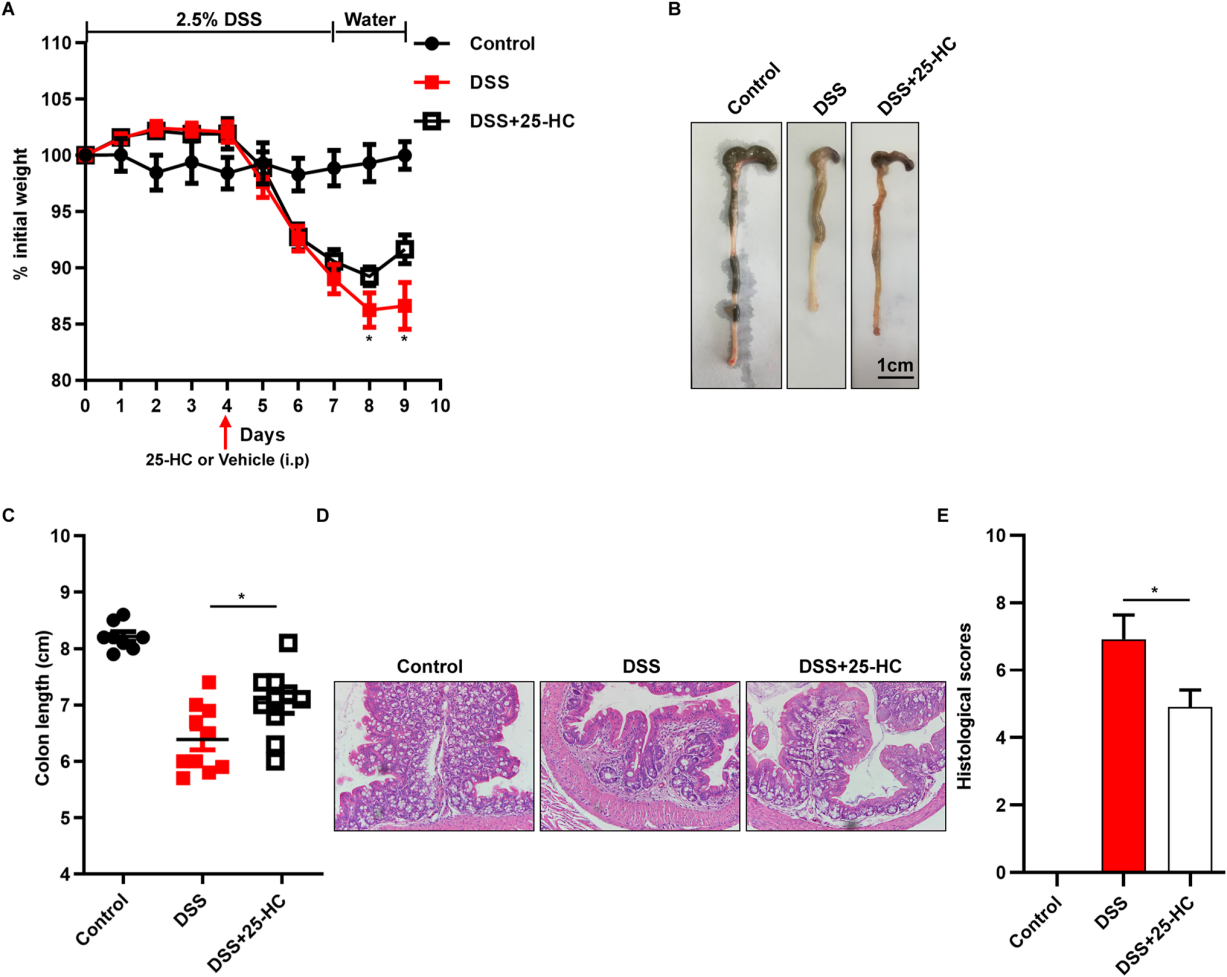
Exogenous 25-HC ameliorated DSS-induced colitis in mice. (**A**) WT mice were administered DSS + 25-HC (20 mg/kg) or DSS + HβCD (DSS) by intraperitoneal injection, and body weight expressed as percent of initial weight. (**B**) Representative images of colons from control, DSS and DSS + 25-HC mice at day 9. (**C**) Quantification of effect of treatment on colon length. (**D**) Hematoxylin and eosin staining of colon sections from control, DSS and DSS + 25-HC at the indicated times. (**E**) Histological scores were determined as described in the section on Material and methods. Scale bars, 50 µm, six mice per group, *p < 0.05, **p < 0.01. Similar results were obtained in three independent experiments with 8–10 mice per group.

The previous results suggest that *Ch25h*^−/−^ mice are highly susceptible in experimental colitis and that, we then reasoned that administration of exogenous 25-HC might further attenuate colitic damage. Four days after the initiation of 2.5% DSS administered through drinking water, WT mice were divided into two groups with a parity criterion of the severity of disease symptoms to obtain two groups with similar average disease severity. For this purpose, we treated WT colitic mice (DSS-treated mice) with 25-HC or the vehicle, 2-hydroxypropyl-β-cyclodextrin (HβCD) by intraperitoneal injection beginning the 4th of DSS treatment and for 2 additional days. Compared to vehicle-treated mice, 25-HC-treated mice showed significant protection from DSS-induced colitis based on body weight loss, colon length and histopathology (Fig. 4A,B). 25-HC treated mice showed significantly lower weight loss beginning on day 4 and lasting throughout the duration of the intraperitoneal injections of 25-HC, as well as a significantly increased colon length on day 9 (Fig. 4C). Consistent with the ameliorated clinical signs, histological analysis confirmed that the administration of 25-HC significantly reduced the DSS-induced colon damage (Fig. 4D,E). Taken together, these results suggest that 25-HC ameliorates the clinical parameters and histological damage of DSS-induced colitis.

### Supplementation with exogenous 25-HC decreased the secretion of pro-inflammatory cytokines and preserved gut barrier functions

**Figure 5:**
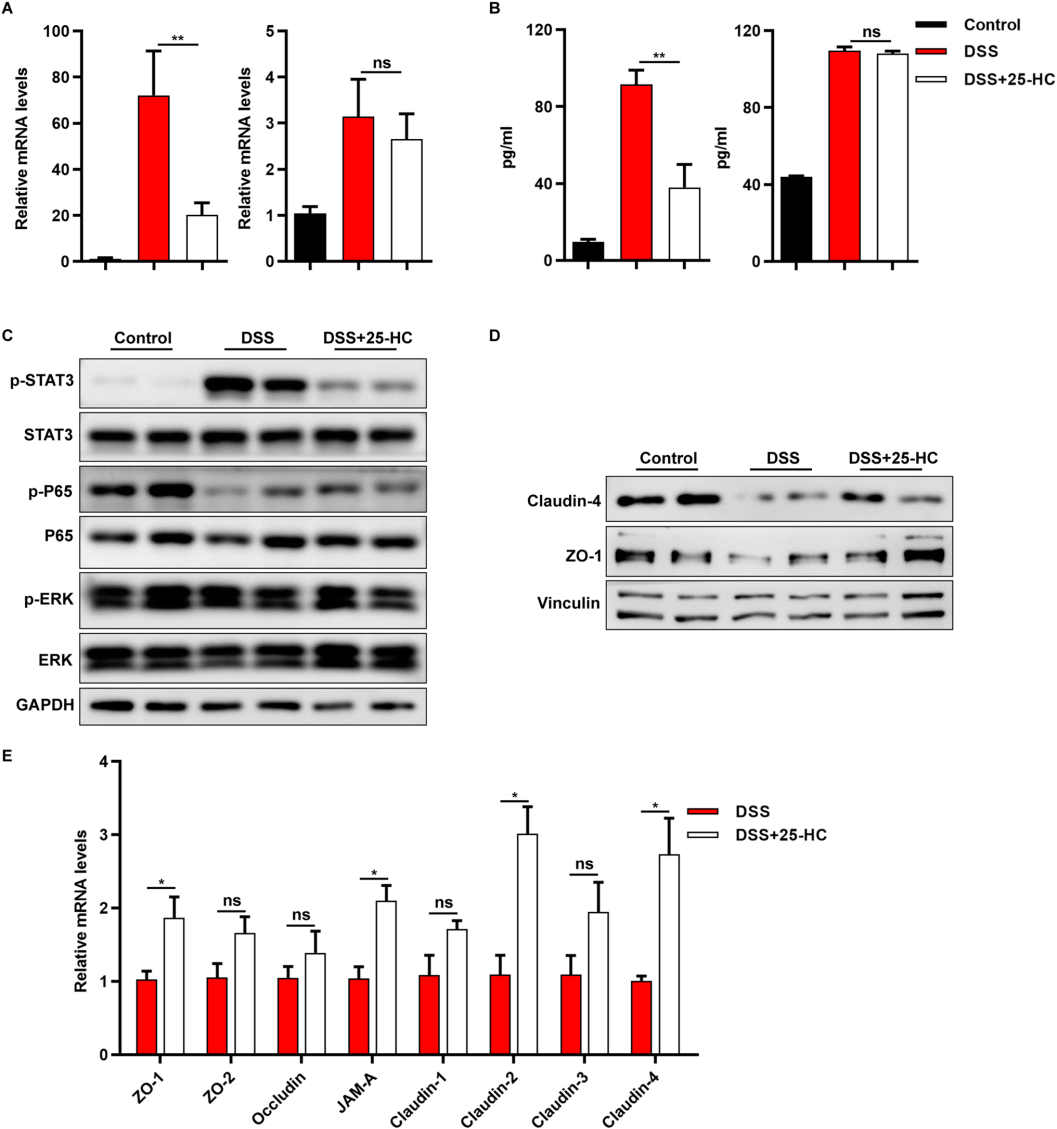
Exogenous 25-HC ameliorated DSS-induced inflammation of the colonic mucosa and repressed the STAT3 signaling pathway. (**A**) The mRNA expression levels of IL-6 and TNF-α in colon tissue were measured by quantitative PCR, and normalized against GAPDH. (**B**) The serum levels of the pro-inflammatory IL-6 and TNF-α were measured by ELISA. (**C**) Western blotting results of STAT3, ERK, P65 in colon. (**D**) The mRNA expression levels of ZO-1, ZO-2, Occludin, JAM-A, Claudin-1, Claudin-2, Claudin-3 and Claudin-4 in colon tissue were measured by quantitative PCR. (**E**) Western blotting results of ZO-1 and Claudin-4 in colon.*p < 0.05, **p < 0.01. The data are expressed as the mean ± S.E.M. Similar results were obtained in three independent experiments with 6–8 mice per group. Full-length blots are included in the supplementary information.

We next investigated whether 25-HC treatment influences the secretion of pro-inflammatory cytokines in DSS-induced colitis. We measured the mRNA expression levels of several pro-inflammatory cytokines including IL-6 and TNF-α, in colon cells from mice on day 9. As shown in Fig. 5, 25-HC treatment significantly decreased the mRNA levels of IL-6, but not of TNF-α, in DSS-induced mice (Fig. 5A). We next detected the serum concentrations of serum IL-6 and TNF-α, 25-HC treatment also significantly decreased the serum levels of IL-6 in DSS-induced mice (Fig. 5B). We then examined the levels of phosphorylated STAT3 by western blot in colon tissue derived from the control group, DSS-treated group and 25-HC-treated DSS-induced group. The phosphorylation level of STAT3 was significantly decreased in 25-HC-treated DSS-induced mice compared to DSS-treated mice, but the phosphorylation of ERK and P65 were not altered in three groups (Fig. 5C). We also treated colitic WT and *Ch25h*^−/−^ mice with 25-HC by intraperitoneal injection beginning on the 4th day of DSS treatment and for 2 additional days. There was a slight improvement in *Ch25h*^−/−^ mice, but there was no significant difference (Supplementary Fig. S3A–C).

25-HC-treated mice showed an increased mRNA expression of tight junction genes, including ZO-1, JAM-A, Claudin-2 and Claudin-4 in the colon compared with that shown by WT DSS-treated mice. ZO-2, Occludin, Claudin-1, Claudin-3 and Claudin-4 mRNA levels displayed an increasing trend in 25-HC- and DSS-treated mice compared to DSS-treated mice (Fig. 5D). There was also increased ZO-1 protein expression which was treated with 25-HC (Fig. 5E). In agreement with the alleviation of colitis symptoms, these results suggest that 25-HC administration helps to suppress inflammatory responses and preserve gut barrier function in mice.

### 25-HC induced tight junction proteins by regulating ATF3

**Figure 6:**
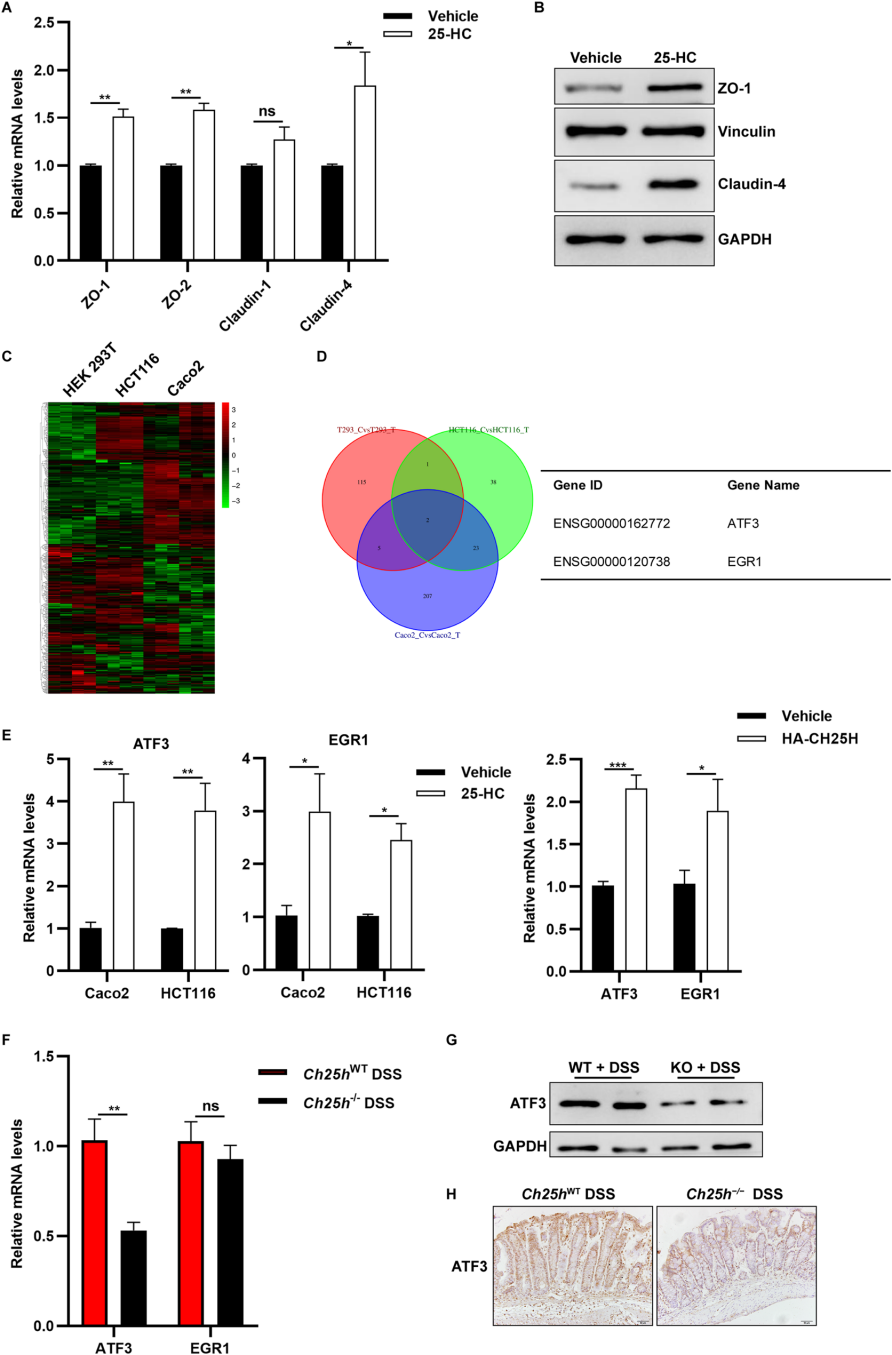
25-HC induced tight junction proteins. (**A**) The fold changes in mRNA levels of ZO-1, ZO-2, Claudin-1 and Claudin-4 in Caco2 cells treated with 25-HC (50 µM) and vehicle were determined by quantitative PCR method. (**B**) 25-HC induced protein expression of ZO-1 and Claudin-4 in Caco2 cells were determined by western blotting. (**C**) A genome-wide RNA-seq heat map showing differentially expressed genes. (**D**) Commonly altered genes (FDR < 0.05, twofold change) across three cell lines after treatment with 25-HC in vitro. Listed are the two genes commonly altered by 25-HC across all three states as determined by RNA-seq. **E.** The mRNA levels of ATF3 and EGR1 were detected in Caco2 cells, HCT116 cells and HEK293T cells. (**F**–**H**) The ATF3 mRNA and protein expression, ATF3 immunostaining of the colon sections from WT and *Ch25h*^−/−^ mice. Full-length blots are included in the supplementary information.

25-HC showed a protective role against experimental colitis and changed the expression of tight junction genes in *Ch25h*^−/−^ mice. A potential therapeutic avenue for IBD is the ability to increase barrier function^31^. To examine such effects, we cultured the colorectal epithelial cell line Caco2 and observed a significant increase in the expression of the tight junction proteins ZO-1, ZO-2 and Claudin-4 using real-time PCR in 25-HC treated cells (Fig. 6A). The increased levels of ZO-1 and Claudin-4 proteins induced by 25-HC was confirmed by western blotting (Fig. 6B). The same results were observed in another epithelial cell line, HCT116 (Supplementary Fig. S5A–B). Overall, these results suggest that treatment with 25-HC increases the expression of tight junction proteins potentially enhancing the gut barrier integrity.

To further investigate how CH25H affects epithelial cells, RNA sequencing (RNA-seq) analysis identified 237 significantly dysregulated (112 up- and 125 down-regulated) genes in 25-HC treated Caco2 cells, and 64 dysregulated (39 up- and 25 down-regulated) genes in 25-HC treated HCT116 cells (Fig. 6C). Some 123 significantly dysregulated (63 up- and 60 down-regulated) genes in HEK 293T cells transfected with CH25H-HA plasmid (Fig. 6C). Among these dysregulated genes, only 2 genes were commonly induced in all three cell lines: ATF3 and EGR1 (Fig. 6D). We confirmed that ATF3 and EGR1 were induced across all cells as verified by real-time PCR and western blotting (Fig. 6E and Supplementary Fig. S5C).

To determine whether these changes are consistent in mice. We found that only ATF3 was markedly reduced in DSS-induced *Ch25h*^−/−^ mice compared with controls, with no changes with EGR1 (Fig. 6F). Activating transcription factor 3 (ATF3) is a member of the CREB family of transcription factors. ATF3 has been shown to control JNK and STAT signaling to maintain intestinal barrier regeneration^32^. Western blots and inmunohistochemical analysis showed that ATF3 were decreased in *Ch25h*^−/−^ mice (Fig. 6G,H), these findings indicate an essential role of CH25H in maintaining basal levels of ATF3 expression.

## Discussion

In light of a recent RNA-sequencing analysis showing that up-regulation of CH25H in patients with active IBD, while lacking evidence supporting a role of CH25H in intestinal homeostasis and IBD pathogenesis, we have performed in-depth analyses here, using animal models, to reveal how CH25H is acting as a critical regulator of the STATS/IL-6 signaling cascade in intestinal cells. Our findings suggest that CH25H exerts critical effects on maintaining epithelial gut barrier function.

Previous studies showed that the CH25H could play opposing roles, either pro-inflammatory or anti-inflammatory, according to different inflammatory disease models. 25-HC has been shown to induce the release of pro-inflammatory cytokines, such as IL-8 and IL-6 and to amplify the response of TLR3 in airway epithelial cells via NF-κB^33^. It has been reported that 25-HC amplifies inflammatory signalling in bone marrow-derived macrophages (BMDMs) in mice via AP-1 following infection with influenza^14^. 25-HC has been reported to activate the NLRP3 inflammasome to increase the release of IL-1β but not to affect IL-6 in neuroinflammation^34^. Other studies have showed that 25-HC dramatically increases IL-1β in human macrophages, but it decreases the release of IL-6 in LPS-treated cells^35^. However, recent reports have found that 25-HC negatively regulates IL-1β transcription by antagonizing SREBP processing in LPS-treated BMDMs^18^. Here, this study revealed that CH25H participates in the pathophysiology of colitis in mice through its protective and anti-inflammatory roles. This observation is in contrast with the findings of a recent study that showed no effect in DSS-treated *Ch25h*^−/−^ mice^36^. We suspect that there are two main explanation for this inconsistency. One explanation concerns the gender of the mice, male but not female mice were used in this study. The other explanation is that the different microbiota conditions in the animal facilities account for this discrepancy. Another study demonstrated reduced intestinal fibrosis in *Ch25h*^*−/−*^ mice using the DSS-induced chronic colitis model^25^, however, a role for CH25H in IBD has not yet been investigated.

In this study, we found that, compared to WT littermates, mice with *Ch25h* depletion exhibit a higher severity of DSS-induced colitis, including more damage to colonic wall structure and more inflammation. Consistently, *Ch25h*-deficient mice exhibit increased activation of STAT3 and levels of inflammatory cytokines IL-6 in the colon tissue. Based on our results, we can hypothesize that 25-HC is a mediator of the defense mechanism and of the protective and repair responses triggered to minimize and reverse colon injury. The protective and anti-inflammatory effects of exogenous 25-HC were demonstrated in this study. Our results demonstrated that supplementation with exogenous 25-HC further reduces the extent of damage. Mice treated with 25-HC showed lower colon damage and lower expression levels of IL-6 in the colon compared with those shown by untreated colitic mice.

We demonstrated that a lack of CH25H leads to increased susceptibility to DSS-induced colitis, which may in part result from impaired epithelial compensation and regeneration mechanisms as indicated by the diminished expression of the mRNA levels of the tight junction genes ZO-1, ZO-2, Claudin-1, Claudin-2 and Claudin-3 in *Ch25h*-deficient mice compared with WT mice. Supplementation with exogenous 25-HC in colitic mice ameliorated the effects of colitis and barrier defects. There was a significant increase in the mRNA levels of ZO-1, Claudin-2 and Claudin-4 in mice treated with 25-HC but not in *Ch25h*-deficient mice treated with 25-HC, and this finding was consistent with intestinal epithelial barrier function (Supplementary Fig. S3). Furthermore, recent work demonstrated the STAT3-dependent regulation expression of ZO-1, Occludin and associated tight junction genes^37^. Thus, we propose that 25-HC has a protective function against intestinal inflammation by helping to maintain epithelial gut barrier integrity and regeneration.

Previous studies have shown that ATF3 acts a stress-inducible gene, which has been described as a regulatory role in macrophage transcriptional response to inflammatory stimuli. Under loss of ATF3, the levels of CH25H and 25-HC are increased, and ATF3 weekly binds to CH25H promoter region^38^. In our study, the expression of ATF3 in colorectal epithelial cell lines Caco2 and HCT116 was increased by 25-HC treatment, and the level of ATF3 was also increased by transfection of CH25H plasmid in HEK 293T cells. Given these findings, in the absence of CH25H mice, the level of ATF3 is decreased in DSS-induced colitis. Our findings establish that, the transcript levels of ATF3 is closely related to CH25H in both the basal state and in the induced state. Correlated to this, several studies have linked ATF3 to epithelial barrier function. In a mouse model, ATF3 was shown to involve in IL-6-mediated STAT3 activation and maintain epithelial barrier function^32^. Loss of ATF3 enhances intestinal permeability and susceptibility to colitis. Above these results, we predict that CH25H might protect intestine from DSS-induced colitis by promoting ATF3 expression.

In conclusion, our data demonstrate that exogenous 25-HC shows a limited protective capacity against tissue damage during colitis. We have demonstrated that CH25H appears to play an important role in maintaining epithelial gut barrier integrity, potentially by influencing the expression of tight junction proteins. Then, it triggers a series of inflammatory responses, including IL-6 release and the activation of STAT3. Further considering the diversity of human IBD, more research is needed to address the potential mechanism underlying the contribution of CH25H to the development of inflammatory disease.

## Materials and methods

### Mice

The Ethics Committee at Nanjing University approved all mouse studies (Confirmation Number: AP#FXY08). Mice were maintained in an Association for Assessment and Accreditation of Laboratory Animal Care International-accredited SPF animal facility (Nanjing Biomedical Research Institute of Nanjing University). All protocols involving animals were conducted in accordance with the Institutional Animal Care and Use Committee of the Model Animal Research Center, Nanjing University. All the experiments were carried out according to the international guidelines. *Ch25h*^−/−^ mice were generated by CRISPR/Cas9 at the Model Animal Research Center (MARC) of Nanjing University. Two sgRNA oligos were synthesized and annealed to the pUC57-sgRNA construct. In vitro transcription was performed as described previously^39^. The Cas9 mRNA and sgRNA were injected in the background of C57BL6/J.

The potential off-target sites for each sgRNA were analyzed by Optimized CRISPR Design (https://crispr.mit.edu/). The top 5 potential off-target sites for each sgRNA were selected for T7EN1 assay.

### Induction of DSS colitis and 25-HC administration

To induce colitis, 8- to 10-week-old WT and *Ch25h*^−/−^ male mice were fed with 2.5% DSS (MP Biomedicals, molecular weight, 36,000–50,000) solution to drink for 7 days followed by drinking water alone for 2 days. Mice were weighed daily and scored for colitis-associated symptoms.

Four days after starting DSS, C57BL6/J mice were divided into two groups, mice were administered DSS + 25-HC (20 mg/kg, Sigma) or DSS + vehicle control (HβCD, Sigma) by intraperitoneal injection daily, from the 4th day of DSS treatment and for 2 additional days. At day 10, mice were killed by CO_2_ asphyxiation and colon length was measured.

### Histopathological analysis

Sections (5 µm thick) from 4% formalin solution and paraffin-embedded colons were placed onto glass slides, and stained with Hematoxylin and eosin (H&E) for histopathological analysis. The histopathological score of colonic inflammation was presented as previously described^40^.

### RNA isolation and quantitative PCR

Total RNA were isolated with TRIzol reagent (Vazyme, RC101). cDNAs were synthesized by PrimeScript RT reagent Kit with gDNA Eraser (TaKaRa, Cat #RR047A). Amplification was performed with ChamQ SYBR qPCR Master Mix (Vazyme, Q311-01/02). The mRNA level of each target gene was normalized to *Gapdh*. Each reaction was performed in triplicate. Data analysis was performed using the ΔΔCt method. Primers were listed in supplementary (Supplementary Table S1).

### Western blotting

Total protein from colon tissues and cells were extracted by RIPA buffer and separated by SDS-PAGE then transferred onto polyvinylidene fluoride membrane. The membrane was blocked in 5% non-fat dry milk or bovine serum albumin for 1 h and then blotted with anti-p-STAT3 (Cell Signaling, 39131L), anti-STAT3 (Cell Signaling, #9139), anti-P65 (Cell Signaling, #8242T), anti-p-P65 (Cell Signaling, #3033S), anti-ERK (Cell Signaling, #9102) anti-p-ERK (Cell Signaling, #9106L), anti-ZO-1 (Millipore, #2153084), anti-GAPDH (Santa Cruz, sc-32233), anti-Claudin-4 (Abclonal, A2947), anti-Vinculin (Cell Signaling, 4650S). Secondary antibody was incubated for 1 h at room temperature. The proteins were detected using Tanon High-sig ECL Western Blotting Substrate (Tanon, #180-501) by Amersham Imager 600 (GE Healthcare). GAPDH was used as loading control.

### IHC staining

Colon tissue sections were deparaffinated with xylene and rehydrated in alcohol. Antigen retrieval was achieved in citrate buffer (PH 6.0) at 99 °C for 20 min, followed by the incubation with primary antibody, anti-Ki67 (Bioword, A25479, 1:500), anti-ZO-1 (Sigma-Aldrich, AB2272, 1:500), anti-ATF3 (Santa Cruz, sc-188, 1:200). Then the slides were stained with the DAB kit (Maixin Bi, DAB-2031) and counterstained with hematoxylin and mounted.

### Quantification of released TNF-α and IL-6

TNF-α and IL-6 in mouse serum were determined using mouse TNF-α and IL-6 enzyme-linked immunosorbent assay kit (4A Biotech, China) according to the manufacturer’s instruction.

### Leukocyte preparation and flow cytometry

Leukocytes from colons, spleen and blood were isolated as described in previously described^41^. Isolated leukocytes were collected, washed, and stained with antibody specific for mouse CD45, CD3, CD4, CD8, F4/80 and CD11b from Biolegend, then analyzed by FACS. Briefly, colon tissues were removed and washed with ice-cold phosphate-buffered saline (PBS), then cut into fragment about 1 cm in length before transferred into DMEM containing 1 mM dithiothreitol (DTT) and 1 mM EDTA at 37 °C for 1 h with shaking. The supernatant was collected and stored at 4 °C. The remaining tissue pieces were treated with collagenase (Sigma, C5138) and DNase I (Sigma, 11284932001) at 37 °C for 1 h with shaking. After incubation, vortex the cell solution and pass through a 40 µM cell strainer set over a 50 ml tube. Following centrifugation, cells were aspirated and washed with FACS buffer (1% fetal bovine serum in PBS), resuspended in FACS buffer and incubated with antibodies. Spleen and blood leukocytes were prepared as described previously.

### Cell culture

HEK 293T cells, Caco2 cells and HCT116 cells were cultured in DMEM-high glucose (HyClone, SH30022.01), supplemented with 10% FBS (Gibco, 10099-141), and 1% penicillin/streptomycin in a 5% CO_2_, 95% humidity environment at 37 °C. HEK 293T cells were transfected with vector or HA-CH25H, after 24 h, the cells were harvested and lysed, and then the whole cell extracted were subjected to western blotting or quantitative PCR. Caco2 cells and HCT116 cells were incubated with 25-HC (50 µM) for 24 h, cells were lysed for extraction of proteins and RNA.

### RNA-seq

HEK 293T cells, Caco2 cells and HCT116 cells’ RNA quality was checked using the Agilent 2,100 Bioanalyzer (RIN > 7 for all samples). Barcoded sequencing libraries were then generated using 1 µg of RNA with rRNA depletion method to construct chain specific transcriptome libraries (Vazyme, NR604) according to the manufacturer’s instruction.

### Statistical analysis

All experiments data were presented as means ± standard deviation (SD). Statistical analysis of differences between two groups were performed using GraphPad Prisms 6.0 software. Data are presented as the mean ± S.E.M. *P* value less than 0.05 were considered statistically significant.

## Supplementary information

Supplementary information.

## Abbreviations

25-HC: 25-Hydroxycholesterol
IBD: Inflammatory bowel disease
DSS: Dextran sulfate sodium
CD: Corhn’s disease
UC: Ulcerative colitis
WT: Wild type
CH25H: Cholesterol 25-hydroxylase
EAE: Experimental autoimmune encephalomyelitis
HβCD: 2-Hydroxypropyl-β-cyclodextrin
BMDMs: Bone marrow-derived macrophages
