# Supplementary material for: Cholesterol 25-hydroxylase protects against experimental colitis in mice by modulating epithelial gut barrier function
Source: Sci Rep. 2020 Aug 28;10:14246. doi: 10.1038/s41598-020-71198-1 (PMC7455728; doi:10.1038/s41598-020-71198-1)
Supplement: Supplementary file 1 — Supplementary information. [file 41598_2020_71198_MOESM1_ESM.pdf]

Cholesterol 25-hydroxylase protects against experimental colitis in mice by modulating epithelial gut barrier function

Na Sheng*[1‡], Zhongnan Ma[1‡], Yi Zhou[2], Juan Xu[3], Yan Gao[1], Xin-Yuan Fu[1, 2]*

[1]The State Key Laboratory of Pharmaceutical Biotechnology and MOE Key Laboratory of Model Animals for Disease Study, Model Animal Research Center, Nanjing University, Nanjing 210061, China.

[2]State Key Laboratory of Biotherapy/Collaborative Innovation Center for Biotherapy, West China Hospital, Sichuan University, Chengdu, Sichuan 610041, China.

[3]Deparment of Gynecology, Women's Hospital of Nanjing Medical University (Nanjing Maternity and Child Health Care Hospital), Nanjing 210004, China.

[‡] Both authors contributed equally to this work.

* Correspondence to: Na Sheng and Xin-Yuan Fu, Model Animal Research Center of Nanjing University, Nanjing 210061, China. Tel.:+86 02558641523; Email: shengna@nicemice.cn and fuxy@nicemice.cn / fulabnus2012@yahoo.com

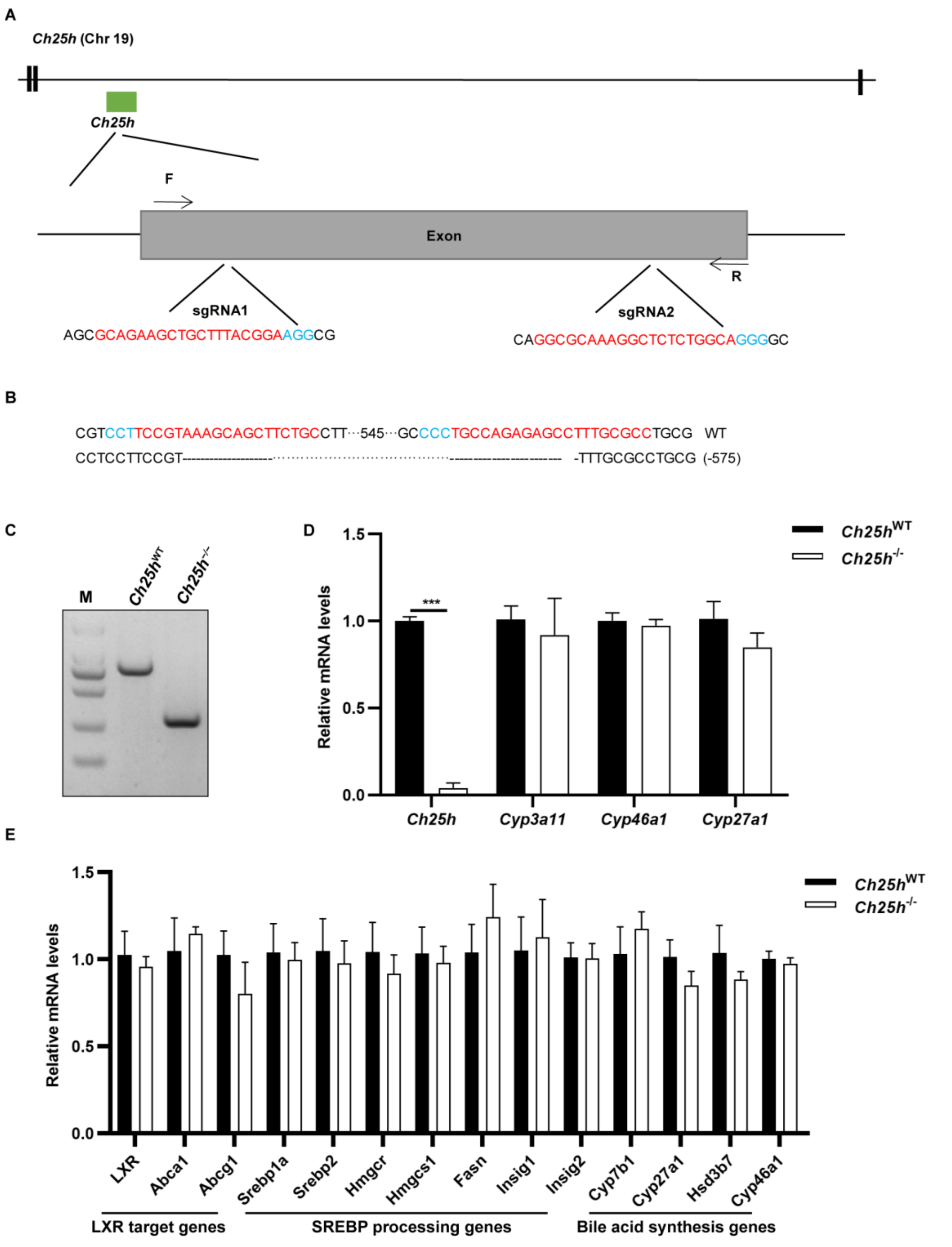

Figure S1. CRISPR/Cas9 mediated *Ch25h* gene knockout in mouse. A. Schematic diagram of *Ch25h* sgRNAs. *Ch25h* gene is in green rectangle. Two sgRNAs were designed to target at head and tail of *Ch25h* exons. **B.** The sequencing results showed fragment deletion in *Ch25h* deleted mice. **C.** Genotyping of WT and knockout mice by PCR. **D.** *Ch25h, Cyp3a11, Cyp46a1 and Cyp27a1* mRNA expression were highly measured in mice. **E.** The mRNA expression levels of LXR target genes, SREBP processing genes and bile acid synthesis genes in colon tissue were measured by quantitative PCR, and normalized against GAPDH (n=4).

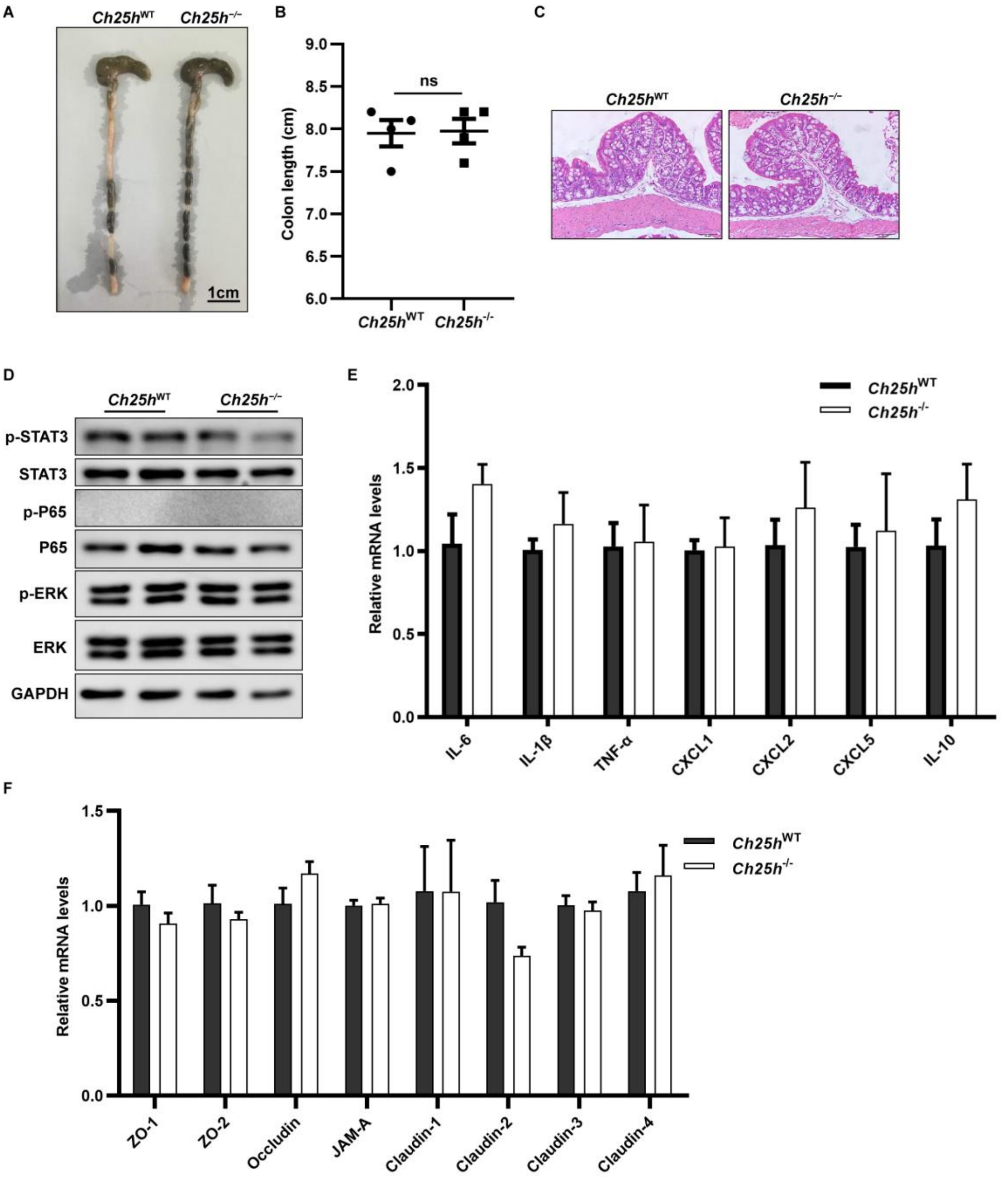

Figure S2. Characterization of intestinal epithelial deletion of *Ch25h* under basal condition. A. Representative images and quantification of colons from WT and *Ch25h*$^{-/-}$ mice**. B.** HE staining demonstrated normal histology in WT and *Ch25h*$^{-/-}$ mice at baseline. **C.** Western blot results of STAT3, ERK, NF-κB in colon (n=4). **D.** The mRNA expression levels of IL-6, IL-1β, TNF-α, CXCL1, CXCL2 and CXCL5 in colon tissue were measured by quantitative PCR, and normalized against GAPDH. **E.** The mRNA expression levels of ZO-1, ZO-2, Occludin, JAM-A, Claudin-1, Claudin-2, Claudin-3 and Claudin-4 in colon tissue were measured by quantitative PCR. Scale bars, 50μm, *$p<0.05$, ** $p<0.01$. Similar results were obtained in three independent experiments with 4-6 mice per group.

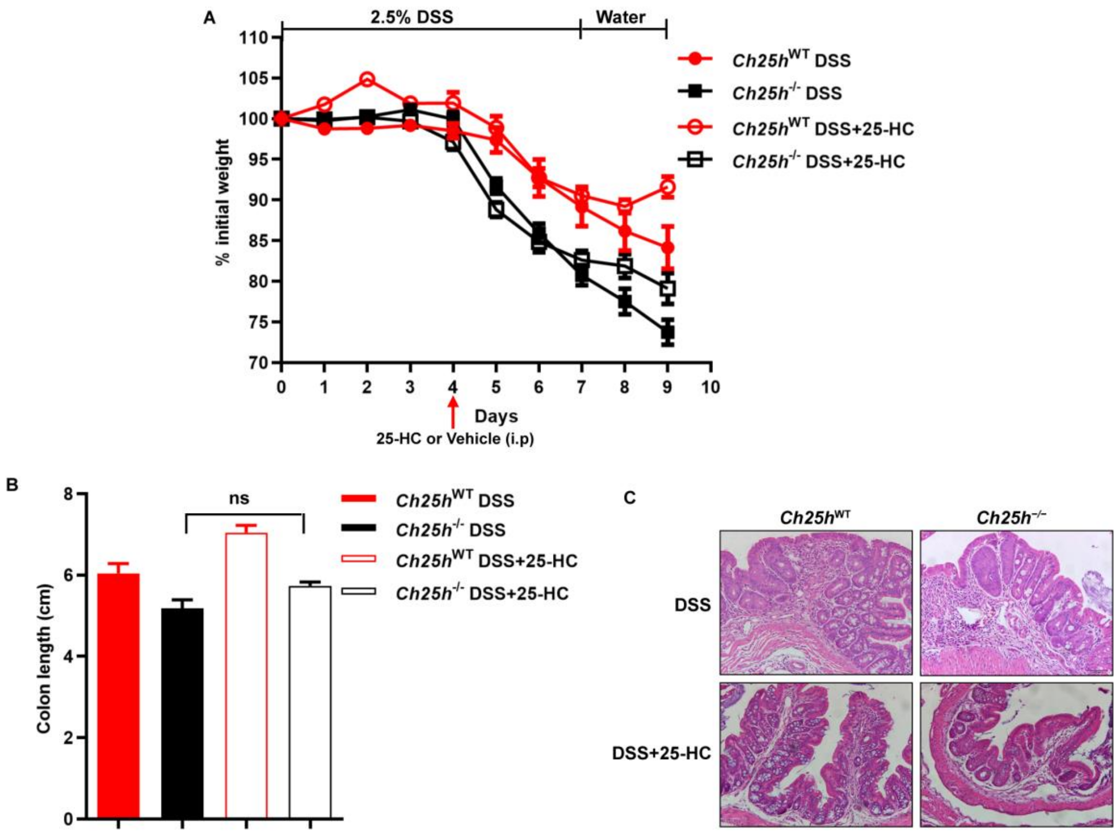

Figure S3. Exogenous 25-HC with DSS-induced colitis in WT and *Ch25h*$^{-/-}$ mice. **A.** WT and *Ch25h*$^{-/-}$ mice were administered DSS + 25-HC (20mg/kg) or DSS + vehicle control (HβCD) by intraperitoneal injection, and body weight expressed as percent of initial weight. **B.** Quantification of effect of treatment on colon length. **C.** Hematoxylin and eosin staining of colon sections from DSS + 25-HC or DSS + vehicle mice at the indicated times. Scale bars, 50μm, six mice per group.

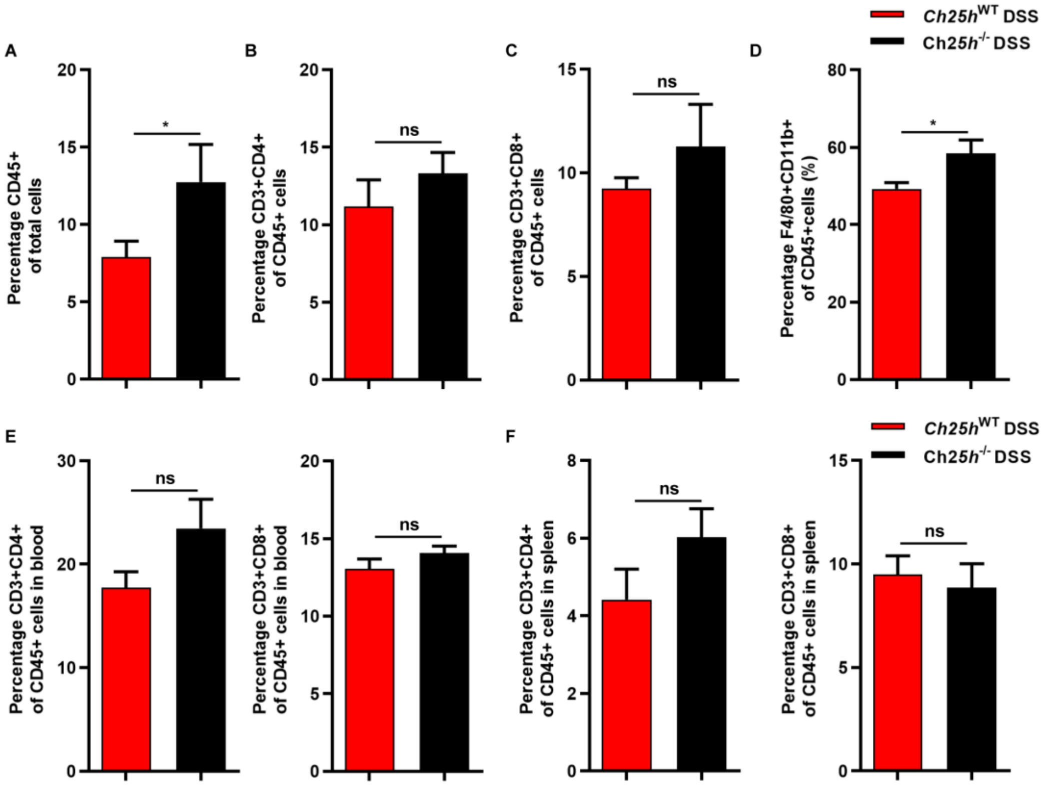

Figure S4. *Ch25h* deficiency exacerbated on the colonic recruitment of inflammation cells following DSS exposure. Colon, spleen and blood cells were prepared from enzymatically digested colon tissue, then were stained with CD45, CD3, CD4, CD8, F4/80 and CD11b antibody and analyzed by cytometry. **A.** The percentage of $CD45^{+}$ cells in a single-cell suspension prepared from enzymatically digested colon tissue. **B.** The percentage of $CD3^{+}CD4^{+}$ in a single-cell suspension prepared from enzymatically digested colon tissue. **C.** The percentage of $CD3^{+}CD8^{+}$ in a single-cell suspension prepared from enzymatically digested colon tissue. **D.** The percentage $F4/80^{+}$ $CD11b^{+}$ of $CD45^{+}$ cells in a single-cell suspension prepared from enzymatically digested colon tissue. **E.** The percentages of T cells in a single-cell suspension prepared from enzymatically digested blood tissue. **F.** The percentages of T cells in a single-cell suspension prepared from enzymatically digested spleen tissue. *$p<0.05$, * * $p<0.01$. The data are expressed as the mean ± S.E.M. Similar results were obtained in three independent experiments with 6-8 mice per group.

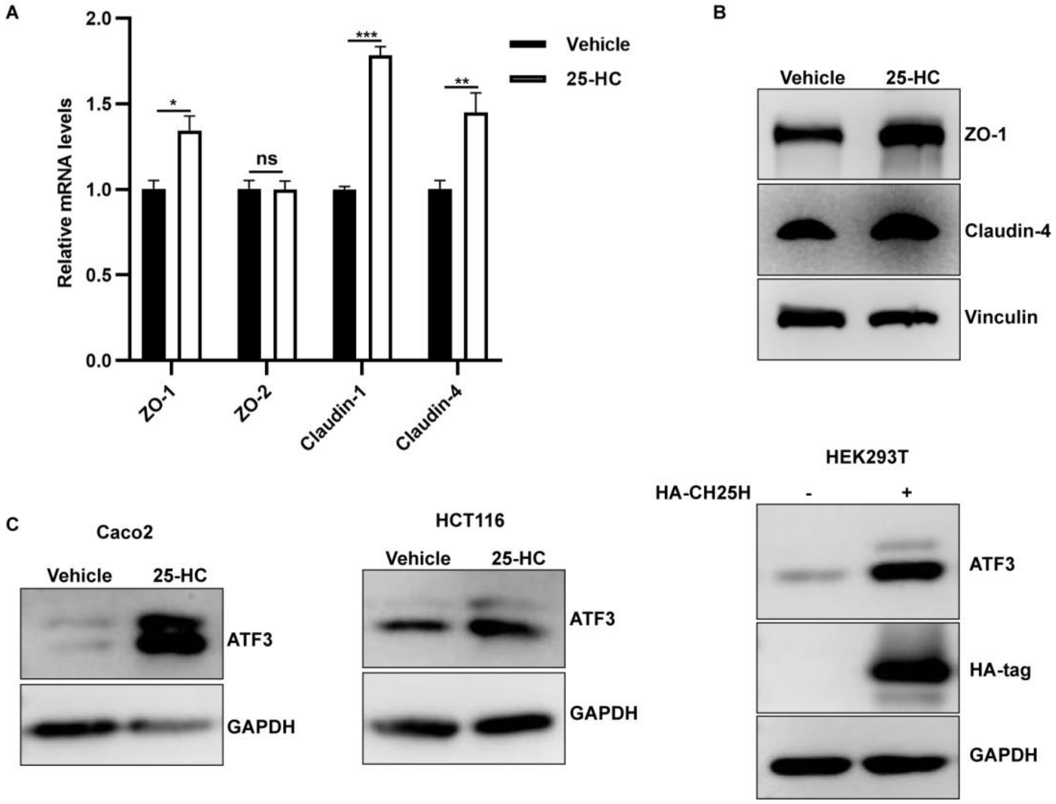

Figure S5. 25-HC induced tight junction proteins. A. The fold changes in mRNA levels of ZO-1, ZO-2, Occludin and Claudin-4 in HCT116 cells were determined by quantitative PCR method. **B.** 25-HC induced protein expression of ZO-1 and Claudin-4 in HCT116 cells were determined by western blot and quantified by Image J software. **C.** The protein levels of ATF3 were detected in Caco2 cells, HCT116 cells and HEK293T cells.

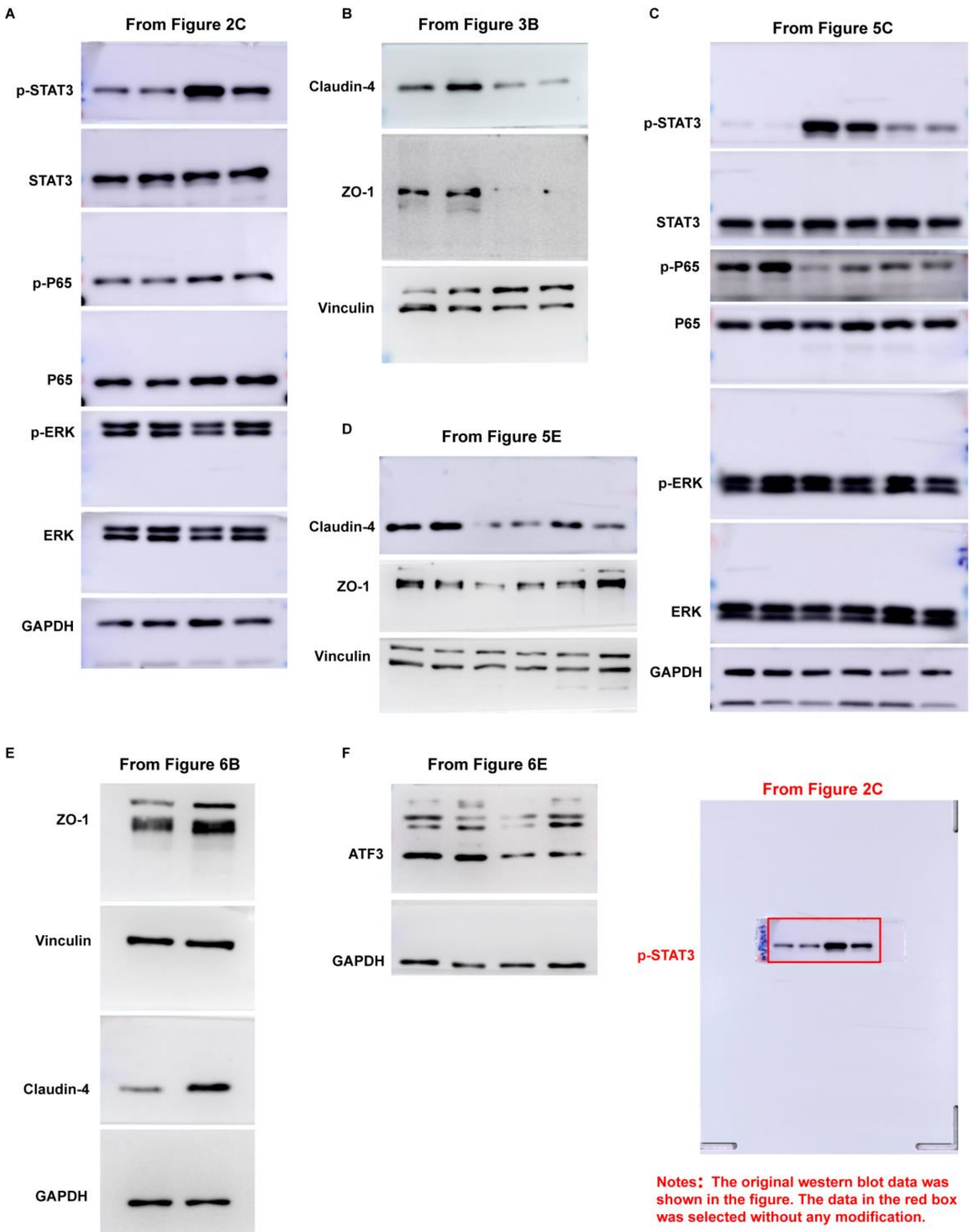

Figure S6. Original panel for each western blot.

G

From Supplementary Figure 1C

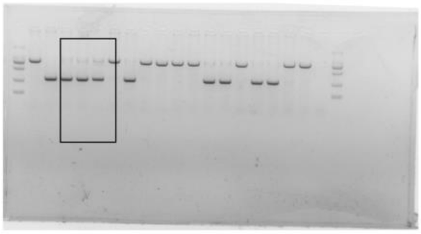

H

From Supplementary Figure 2C

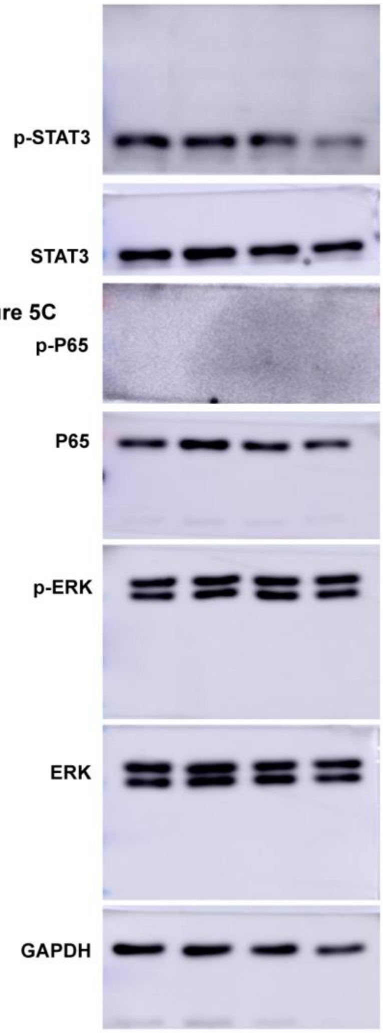

I

From Supplementary Figure 5B

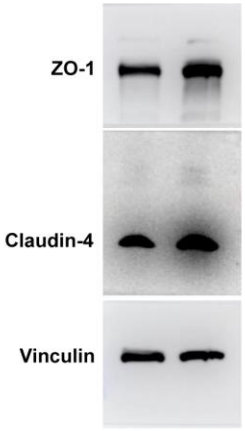

J

From Supplementary Figure 5C

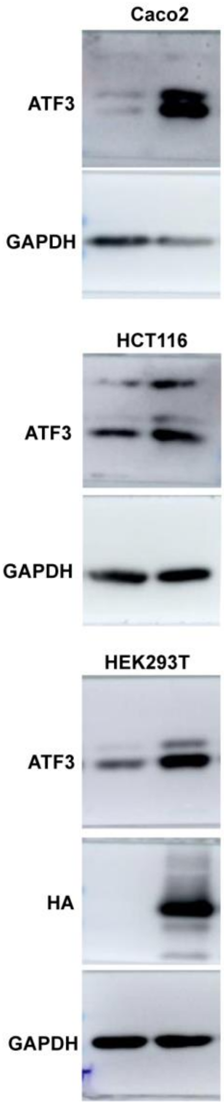

Figure S6. Original panel for each western blot.

Table S1. Sequence of primers used

	Forward	Reverse
Mouse IL-6	CCGGAGAGGAGACTTCACAG	GGAAATTGGGGTAGGAAGGA
Mouse TNF-α	TCCCAGGTTCTCTTCAAGGGA	GGTGAGGAGCACGTAGTCGG
Mouse IL-1β	GCAACTGTTCCTGAACTCAACT	ATCTTTTGGGGTCCGTCAACT
Mouse CXCL1	ACTGCACCCAAACCGAAGTC	TGGGGACACCTTTTAGCATCTT
Mouse CXCL2	CCAACCACCAGGCTACAGG	GCGTCACACTCAAGCTCTG
Mouse CXCL5	GTTCCATCTCGCCATTCATGC	GCGGCTATGACTGAGGAAGG
Mouse Occludin	TGAAAGTCCACCTCCTTACAGA	CCGGATAAAAAGAGTACGCTGG
Mouse ZO-1	GCTTTAGCGAACAGAAGGAGC	TTCATTTTTCCGAGACTTCACCA
Mouse Claudin-1	TGCCCCAGTGGAAGATTTACT	CTTTGCGAAACGCAGGACAT
Mouse Claudin-2	CAACTGGTGGGCTACATCCTA	ATCCAGAGGCCCTTGGAAAAG
Mouse Claudin-3	ACCAACTGCGTACAAGACGAG	CGGGCACCAACGGGTTATAG
Mouse Claudin-4	ATGGCGTCTATGGGACTACAG	GAGCGCACAACTCAGGATG
Mouse ZO-2	GTTTGCCGTTCAGCAGCTTAG	CTTCAAAACCTCGGTCGTCAT
Mouse JAM-A	TCTCTTCACGTCTATGATCCTGG	TTTGATGGACTCGTTCTCGGG
Mouse ATF3	TTTGCTAACCTGACACCCTTTG	AGAGGACATCCGATGGCAGA
Mouse EGR1	TCGGCTCCTTTCCTCACTCA	CTCATAGGGTTGTTCGCTCGG
Mouse Gapdh	AACGGGAAGCCCATCACC	CAGCCTTGGCAGCACCAG
Mouse LXR	GCCTGGGAATGGTTCTCCTC	AGATGACCACGATGTAGGCAG
Mouse Abca1	AAAACCGCAGACATCCTTCAG	CATACCGAAACTCGTTCACCC
Mouse Abcg1	TTCCCCTGGAGATGAGTGT	CAGTAGGCCACAGGGAACAT
Mouse Srebp1a	TTGGCACCTGGGCTGCT	GCGCCATGGACGAGCTG
Mouse Srebp2	CTTGACTTCCTTGCTGCA	GCGTGAGTGTGGGCGAATC
Mouse Hmgcr	AGCTTGCCCGAATTGTATGTG	TCTGTTGTGAACCATGTGACTTC
Mouse Hmgcs1	GGAAGCCTTTGGGGACGTTA	ACACTCCAACCCTCTTCCCT
Mouse Fasn	TTGCTGGCACTACAGAATGC	AACAGCCTCAGAGCGACAAT
Mouse Insig1	CACGACCACGTCTGGAACTAT	CCAAATGAGAAGAGCACTAGGCT
Mouse Insig2	TTGGGCTGTTGTACCCCTG	TGGCGTGATTTATACCCACGA
Mouse Cyp7b1	GGAGCCACGACCCTAGATG	TGCCAAGATAAGGAAGCCAAC
Mouse Cyp27a1	GCACAGGAGAGTACGGAGG	CGGGCAAGTGCAGCACATA
Mouse Cyp46a1	AGCCGCTATGAGCACATCC	CCATACTTCTTAGCCCAATCCAG
Mouse Hsd3b7	AGGCCAGTCCAAAGACCATC	TGCTCGTGTAGACCAGGTACT
Mouse Cyp3a11	GGCAGCATTGATCCTTATG	AAGAACTCCTTGAGGGAGAC
Human ZO-1	CAACATACAGTGACGCTTCACA	CACTATTGACGTTTCCCCACTC
Human ZO-2	GGGAAGGTCGCTGCTATTGT	CTCTCGCTGTAGCCACTCC
Human Claudin-4	TGGGGCTACAGGTAATGGG	GGTCTGCGAGGTGACAATGTT
Human Claudin-1	CCTCCTGGGAGTGATAGCAAT	GGCAACTAAAATAGCCAGACCT
Human ATF3	CCTCTGCGCTGGAATCAGTC	TTCTTTCTCGTCGCCTCTTTTT
Human EGR1	GGTCAGTGGCCTAGTGAGC	GTGCCGCTGAGTAAATGGGA
Human Gapdh	ACAACTTTGGTATCGTGGAAGG	GCCATCACGCCACAGTTTC
